# Surviving extreme radiation

**DOI:** 10.7554/eLife.100219

**Published:** 2024-07-04

**Authors:** Chaitra Shree Udugere Shivakumara Swamy, Thomas C Boothby

**Affiliations:** 1 Department of Molecular Biology, University of Wyoming Laramie United States

**Keywords:** tardigrades, ionizing radiation, DNA repair, Tardigrade, Other

## Abstract

Tiny animals known as tardigrades use a combination of DNA repair machinery and a novel protein to mend their genome after intense ionizing radiation.

**Related research article** Anoud M, Delagoutte E, Helleu Q, Brion A, Duvernois-Berthet E, As M, Marques X, Lamribet K, Senamaud C, Jourdren L, Adrait A, Heinrich S, Toutirais G, Hamlaoui S, Gropplero G, Giovannini I, Ponger L, Gèze M, Blugeon C, Coute Y, Guidetti R, Rebecchi L, Giovannangeli C, De Cian A, Concordet JP. 2024. Comparative transcriptomics reveal a novel tardigrade specific DNA binding protein induced in response to ionizing radiation. *eLife* **13**:e92621. doi: 10.7554/eLife.92621.

When you think about the toughest animal in the world, you might think of a lion or tiger. But a less well-known contender for this title is a tiny animal known as a tardigrade, which is renowned for surviving extreme conditions. This includes being frozen, heated past the boiling point of water, completely dried out, exposed to the vacuum of outer space, and even being bombarded with extremely high levels of ionizing radiation (Kinchin, 1994; Jönsson, 2019).

How tardigrades endure these extremes is one of the prevailing mysteries of physiology. Now, in eLife, Jean-Paul Concordet and Anne de Cian from the Muséum National d'Histoire Naturelle and colleagues – including Marwan Anoud, Emmanuelle Delagoutte and Quentin Helleu as joint first authors – report new insights into how tardigrades survive exposure to ionizing radiation (Anoud et al., 2024).

Ionizing radiation typically damages cells by causing their DNA to fragment (Téoule, 1987). In the past, it was thought that organisms capable of surviving extreme doses of radiation, like tardigrades, might do so by blocking and preventing the radiation from harming their DNA. A previous report suggests that tardigrades utilize a protein known as Dsup to prevent DNA damage during radiation exposure (Hashimoto et al., 2016). However, Anoud et al. found that tardigrades accumulate the same amount of DNA damage as organisms and cells which are intolerant to radiation, such as human cells grown in a dish (Mohsin Ali et al., 2004). If tardigrades do not survive ionizing radiation by directly blocking DNA damage, how do they endure such an insult?

To investigate, the team (who are based at various institutes in France and Italy) examined three species of tardigrade, using a technique known as RNA sequencing, to see which genes are switched on when exposed to ionizing radiation. They also tested tardigrades exposed to bleomycin – a drug that mimics the effects of radiation by creating double-stranded breaks in DNA.

Anoud et al. found that tardigrades upregulated the expression of genes involved in the DNA repair machinery that is common across many life forms, ranging from single-celled organisms to humans. The DNA damage the tardigrades initially accumulated following radiation or treatment with bleomycin also gradually disappeared after the exposure. Overall, these results strongly suggest that to cope with the DNA damage caused by ionizing radiation, tardigrades mount a robust set of repair mechanisms to help stitch their shattered genome back together.

In addition to seeing that DNA repair machinery is upregulated following ionizing radiation, Anoud et al. identified a new gene only present in tardigrades, which encodes a protein they named TDR1 (short for tardigrade DNA repair protein 1). Further experiments revealed that TDR1 can enter the cell nucleus and bind to DNA. This may be due to conserved portions of TDR1, which are largely positively charged, electrostatically interacting with negatively charged DNA. Moreover, at high concentrations, TDR1 not only binds to DNA, but forms aggregates in a concentration-dependent manner. Finally, Anoud et al. found that introducing the gene for TDR1 into healthy human cells reduced the amount of DNA damage caused by bleomycin, indicating that the TDR1 protein helps with DNA repair (Figure 1).

**Figure 1.**
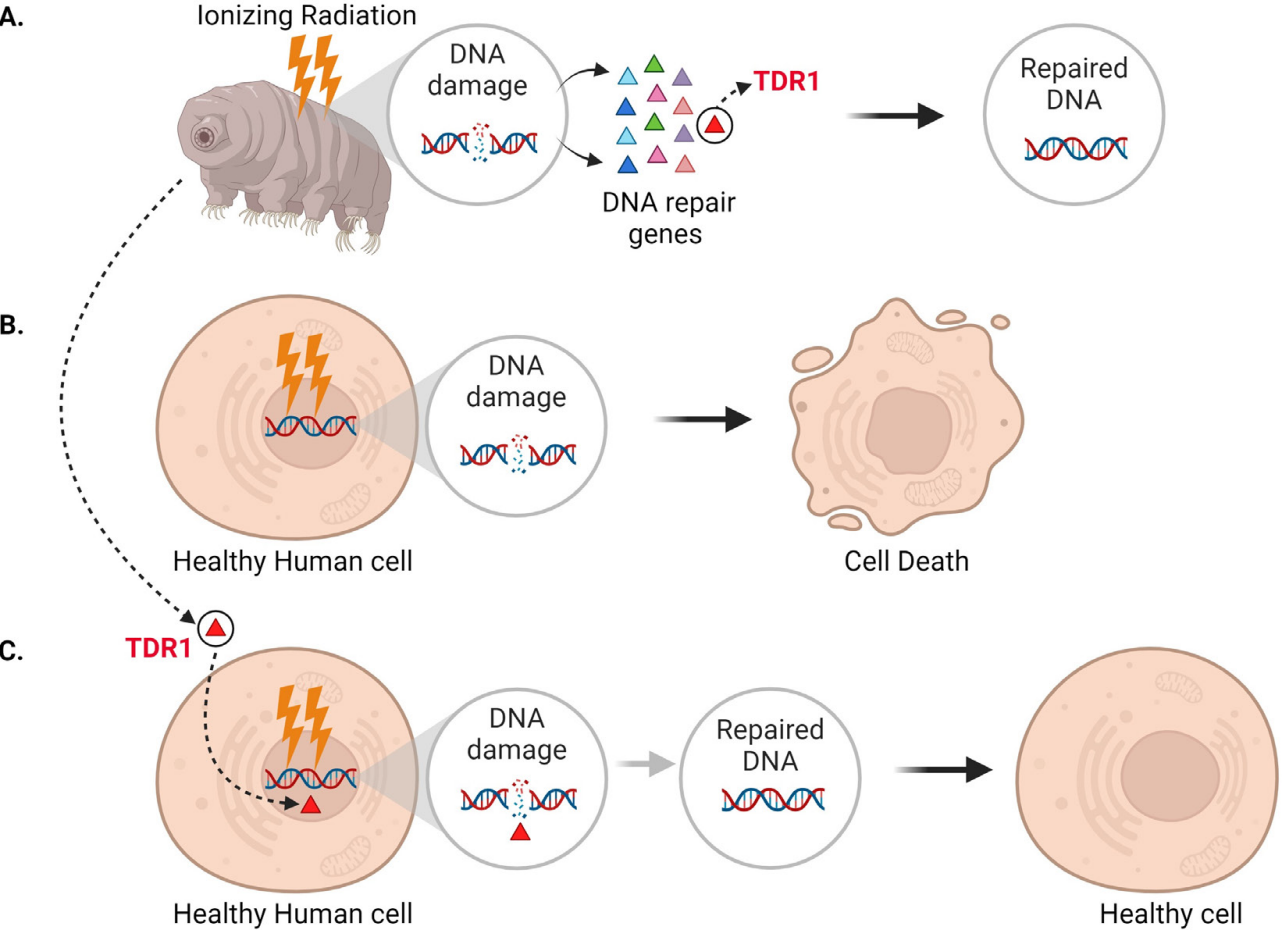
Expressing the tardigrade protein TDR1 in human cells increases DNA damage repair. (**A**) In tardigrades, exposure to ionizing radiation or the drug bleomycin leads to DNA damage, such as double-stranded breaks (circle inset). This switches on various genes (triangles) that repair the genome, including the gene that codes for a protein called TDR1 (red triangle). (**B**) Bleomycin treatment (represented as lightning bolts) also causes double-stranded DNA breaks in human cells. However, these cannot be efficiently repaired, resulting in cell death. (**C**) Anoud et al. found that introducing the gene for TDR1 to the genome of human cells increases their ability to repair DNA damage and their chance of survival.

While it is still unclear exactly how tardigrades fix DNA damage, this study suggests that their ability to survive extreme radiation is related to their strong DNA repair ability, which TDR1 likely plays a crucial role in. Anoud et al. found that TDR1 did not accumulate at DNA damage sites like some other repair proteins (Rothkamm et al., 2015). Instead, they propose that the protein mends DNA by binding to it and forming aggregates which compact the fragmented DNA and help maintain the organization of the damaged genome.

However, more research is needed to fully understand the mechanism responsible for TDR1 and other proteins helping tardigrades to survive ionizing radiation. Ultimately, knowing how these tiny organisms efficiently repair their DNA could lead to novel strategies for protecting human cells from radiation damage, which could benefit cancer treatment and space exploration.
